# IDC-IMPROVE: protocol for a cluster randomised feasibility trial of a care bundle to improve indwelling catheter care (IDC) in residential aged care homes in Australia

**DOI:** 10.1186/s40814-025-01700-x

**Published:** 2025-10-17

**Authors:** Joan OSTASZKIEWICZ, Andrew Simon GILBERT, Caitlin TAY, Elizabeth WATT, David BARRY, Wendy TAYLOR, Jessica CECIL, Rowan COCKERELL, Helen CROWE, Liza LAU, Michael MURRAY, Sigrid NAKREM, Catherine PATERSON, Micah PETERS, Ashlyn SAHAY, Alyson SWEENEY, Janie THOMPSON, Julie WESTAWAY, Wendy BOWER, Juliana CHRISTINA, Alan ZANA, Frances BATCHELOR

**Affiliations:** 1 National Ageing Research Institute, Royal Melbourne Hospital, Parkville, PO Box 2127, VIC 3050 Australia; 2 Faculty of Medicine, Dentistry and Health Sciences, University of Melbourne, Parkville, VIC Australia; 3 Health and Innovation Transformation Centre, Federation University, Ballarat, VIC Australia; 4 Department of Social Inquiry, La Trobe University, Bundoora, VIC Australia; 5 Continence Health Australia, Surrey Hills, VIC Australia; 6 Australian Prostate Centre, North Melbourne, VIC Australia; 7 Austin Health, Heidelberg, VIC Australia; 8 Norwegian University of Science and Technology, Torgarden, Trondheim, Norway; 9 Caring Futures Institute, Flinders University, Adelaide, SA Australia; 10 Central Adelaide Local Health Network, Adelaide, Australia; 11 Rosemary Bryant AO Research Centre, Clinical and Health Sciences, University of South Australia, Adelaide, SA Australia; 12 Australian Nursing and Midwifery Federation (Federal Office), Melbourne, VIC Australia; 13 University of Adelaide, School of Public Health, Health Evidence Synthesis, Recommendations and Impact (HESRI), Adelaide, SA Australia; 14 University of Southern Queensland, Office of the Pro-Vice Chancellor (First Nations Strategy), Ipswich, QLD Australia; 15 The Danish Centre of Systematic Reviews: a Joanna Briggs Institute Centre of Excellence, Department of Clinical Medicine, Aalborg University, Aalborg, Denmark; 16 School of Nursing, Midwifery and Social Sciences, Central Queensland University, Toowoomba, QLD Australia; 17 Darling Downs Hospital and Health Service, Toowoomba, QLD Australia

## Abstract

**Background:**

Indwelling urinary catheters (IDCs) are used by approximately 8% of Australian aged care residents. IDC use is often warranted but entails numerous risks, particularly if used longterm. Risks include catheter-associated urinary tract infections, catheter blockage, catheter leakage, bladder spasm, pain, urethral trauma and haematuria, and increased risk of hospitalisation. The Royal Commission into Aged Care Quality and Safety identified poor quality, unsafe practices related to IDCs in aged care homes. Enhancing the knowledge, confidence, and skills of aged care staff to deliver catheter care for residents with IDCs is fundamentally important. The IDC-IMPROVE project is supporting aged care providers to meet the care needs of people with IDCs in Australian aged care homes, by designing and validating a suite of resources titled the IDC-IMPROVE Catheter Care Bundle.

**Aims:**

This study aims to establish the feasibility of conducting a definitive randomised control trial to evaluate the effects of the IDC-IMPROVE Catheter Care Bundle in aged care homes in Australia.

**Method:**

A multi-centre, facility-level clustered randomised control (cRCT) feasibility trial in 24 aged care homes across Victoria, Queensland, and South Australia. Twelve homes will receive the intervention and 12 will continue usual care. The IDC-IMPROVE Catheter Care Bundle intervention comprises principles for person-centred catheter care, online training for nurses and personal care workers, a practical skills workshop for nurses, a toolkit for managers, and an evidence-to-practice support model. The feasibility of the intervention will be assessed through a mix of qualitative and quantitative methods, including surveys, interviews, and audits. Feasibility outcomes are: (i) The acceptability of the Bundle, (ii) The fidelity of the implementation, (iii) The compatibility of the Bundle with standard aged care home IDC care.

**Discussion:**

By enhancing the knowledge, confidence and skills of the aged care workforce, IDCIMPROVE aims to reduce IDC-related complications. This study will provide insights into the acceptability and implementation of the intervention, informing future large-scale trials and potential policy changes.

**Ethics:**

The study has been approved by Austin Health Human Research Ethics Committee (reg: HREC/107165/Austin-2024) and is registered on the Australian New Zealand Clinical Trials Registry (reg: ACTRN12624001178538p).

**Supplementary Information:**

The online version contains supplementary material available at 10.1186/s40814-025-01700-x.

## Background

Indwelling urinary catheters (IDCs) are devices inserted into the bladder via the urethra or lower abdomen (suprapubic region) and remain in place to aid urine drainage. International estimates suggest between 5 and 8% of people in long-term care homes have an IDC [1–3]. During 2022–2023, 8.4% (15,249) of people receiving government subsidised residential aged care in Australia required some form of catheter care [4].^1^

Although potentially beneficial, IDC use is associated with higher risks of morbidity [5, 6]. IDCs provide a mechanism for bacteria to enter the bladder and therefore people with an IDC are at risk of developing a catheter-associated urinary tract infection (CAUTI). The longer a catheter remains in place, the higher the risk. The European Association of Urological Nurses (EAUN) defines long-term catheterisation as the use of a catheter remaining in situ for ≥ 14 days [7]. CAUTIs can lead to other complications such as encrustation and catheter blockage [8], for which treatment is challenging [9]. CAUTIs and catheter blockages are serious events that may result in presentation to emergency departments [10]. Transferring older people from aged care homes to emergency departments or hospital admission has been associated with further risks, including delirium, increased functional decline, nosocomial infections, and pressure injuries [11, 12].

Projections from the Australian Institute of Health and Welfare (AIHW) and Australian Bureau of Statistics (ABS) show that Australia’s population over 65 will continue to grow substantially over the coming decades, driven by increased life expectancy and lower birth rates [13]. By 2066, it is estimated that around 23% of the population will be over 65. As this demographic shift progresses, age-related health issues—including conditions that may necessitate long-term use of medical devices like IDCs—are expected to rise due to increased healthcare demands among older individuals.

This trend will likely impact the aged care sector, as a significant portion of the older population transition into aged care homes. In these settings, residents often have complex health needs requiring specialised care and skilled staff, particularly for indwelling catheter management and other chronic care interventions. Meeting this demand will necessitate upskilling the workforce through education and training in IDC care and related areas to provide appropriate support and prevent complications associated with long-term use.

The day-to-day care of people with IDCs in aged care homes in Australia is undertaken by personal care workers (PCWs) and registered and enrolled nurses. This workforce is central to the quality and safety of care in residential aged care homes. The Australian Royal Commission into Aged Care Quality and Safety heard evidence of poor quality, unsafe practices related to IDCs and called for interventions to strengthen the capacity of this workforce to care for older Australians [14].

Through the *Improving indwelling urinary catheter care in residential aged care* (IDC-IMPROVE) project, we aim to improve knowledge and skills in catheter care among the residential aged care workforce. Specifically, we aim to support nurses to perform routine uncomplicated catheter exchanges on site; identify, prevent and manage IDC-related problems; and recognise and address the inappropriate use of an IDC.

We have designed and validated a comprehensive, evidence-based intervention titled the *IDC-IMPROVE Catheter Care Bundle* (hereafter referred to as the *Bundle*). The *Bundle* was developed in consultation with key stakeholders and with reference to the most recent comprehensive evidence-based guidelines from the EAUN [7] about urinary catheter management. It has been adapted to suit residential aged care nursing and care staff. The *Bundle* consists of the following:

- Principles for person-centred catheter care
- An online course for registered and enrolled nurses about person-centred IDC care
- An online course for PCWs about person-centred IDC care.
- A catheter care toolkit for managers/senior leaders.

We have also partnered with Continence Health Australia (CHA) to deliver site-based Catheterisation Skills Workshops, where nurses who have completed the online course can develop and practice the skill of inserting and changing IDCs in a safe environment. This article presents the protocol for the next part of the project, a cluster randomised feasibility study of the *Bundle* in residential aged care homes.

This is the first time a trial of a catheter care bundle has been conducted in residential aged care homes in Australia. Investigating feasibility will support refinement of the intervention and the outcome measures for this setting. Moreover, recent research has identified several barriers that impede the involvement of residential aged care homes in research, including staff shortages, systemic and regulatory pressures, under-resourcing, and difficulties selecting unbiased samples [15, 16]. The Australian Royal Commission into Aged Care Quality and Safety recommended increasing the research capacity of Australia’s aged care sector to support system reforms and ongoing quality improvement [14]. It is therefore appropriate and ethical to investigate feasibility via a smaller scale trial before conducting a definitive clinical trial where there are significant risks of failure [17]. The cluster randomised feasibility trial is being conducted in accordance with the extended CONSORT guidelines for feasibility studies [18]. We will use a mixed methods approach with qualitative and quantitative components to determine feasibility.

## Methods

The aims of this study are to determine the feasibility of implementing the *Bundle* in residential aged care homes and to determine the utility and feasibility of a definitive phase III trial to assess its effectiveness. The aims are encompassed by the following five objectives:

1. To determine the acceptability of the *Bundle* to end users in residential aged care homes.
2. To explore the fidelity of the implementation of the *Bundle*.
3. To investigate the compatibility of the *Bundle* with current catheter care practices, procedures and policies.
4. To investigate the internal consistency, construct validity and convergent validity of a scale developed to measure nurses’ knowledge and confidence to provide person-centred IDC care.
5. To gather descriptive pilot data related to rates of IDC-related complications, and staff knowledge and confidence to provide person-centred IDC care.

### Feasibility cRCT

**Fig. 1:**
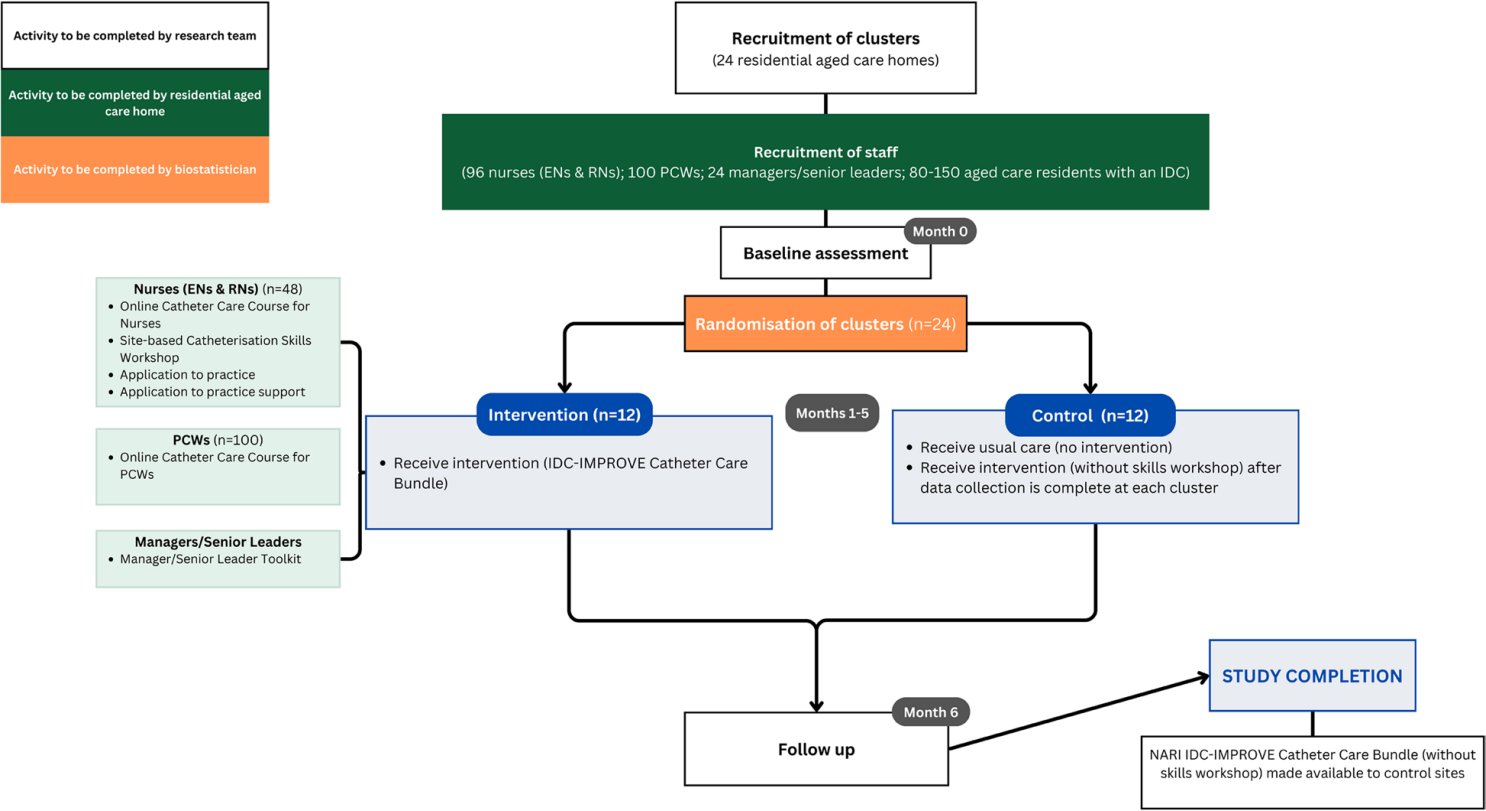
Flow diagram illustrating cluster and participant progression through the NARI IDC-IMPROVE feasibility randomised controlled trial

This is a multi-centre, facility-level cluster randomised control feasibility trial with an embedded process evaluation conducted in residential aged care homes in Australia. After baseline data are collected for all outcome measures, 12 aged care homes will be randomised to receive the intervention and 12 will continue to provide usual practice (control). Each cluster comprises one site (residential aged care home). The control sites are included to assess acceptability of randomisation and fidelity to data collection and to provide a comparator for pilot data. Control sites will receive some elements of the *Bundle* after the trial closes and data collection is completed at their site. Figure 1 provides an overview of the trial.

### Setting

The trial will be conducted over approximately 6 months in 24 aged care homes across three Australian states: Victoria, Queensland, and South Australia. Residential aged care homes provide accommodation and 24-h personal care for older people (aged 65 + years) who can no longer live in their own home [19, 20]. Most aged care in Australia is subsidized and regulated by the Commonwealth government and includes nursing and other general healthcare, but not acute or sub-acute medical care, which falls under state and territory jurisdictions.

The number of sites allows for a diverse sample that reflects the profile of the residential aged care sector in each state. During recruitment, homes will be stratified according to three sizes (small, 60 or fewer residents; medium, 61–100 residents; large, 101 + residents) to ensure a representative sample. We will aim to recruit a diverse sample regarding other site characteristics (e.g. ownership structure, location). In a larger future trial, formally stratifying for these characteristics will be considered.

### Site inclusion/exclusion criteria

The inclusion criteria for sites are the following: (a) an approved residential aged care provider in Victoria, Queensland, or South Australia, (b) has at least 4 nursing staff employed who are available to participate in the study for trial period, and (c) one or more resident with an IDC at time of enrolment. The exclusion criteria are the following: (a) the home is for sale (the potential individual site), (b) does not have a resident with IDC use at the time of enrolment.

### Population

The intervention is multifaceted and targets different residential aged care staff groups, comprising managers/senior leaders, nurses (both Registered (RN) and Enrolled (EN)), and PCWs. This is intended to facilitate multi-level practice change including organisational policy, clinical care, and personal care. All participants must be over 18 years old.

### Participant inclusion/exclusion criteria

#### Nurses

We aim to recruit at least four nurses (RNs and ENs) per site (96 total), as the face-to-face component of the training will be delivered to groups of four. Inclusion criteria require participants to be employed at an enrolled site (permanent or casual); registered to practice in Australia as an EN or RN with the Nursing and Midwifery Board of Australia; are planning to remain employed at the site for the trial period. Exclusion criteria are as follows: the nurse cannot commit to completing the online course about person-centred IDC care and/or attend the Catheterisation Skills Workshops.

#### Personal care workers

We aim to recruit 100 PCWs across intervention and control sites. Inclusion criterion is that the participant plans to be employed at the site during the trial period.

#### Managers and senior leaders

We aim to recruit 1–2 managers or senior leaders from each intervention and control site. To participate, they must have responsibilities in managing or coordinating the overall quality of care (e.g. infection control, staff development) and anticipated to remain employed at the site during the trial period.

#### Residents

All residents at participating sites who have IDCs and are capable of providing informed consent, or whose proxy can provide consent, will be invited to participate. Residents will not be receiving any treatment or completing any measures. Consent is being sought for permission to extract data from residents’ medical records.

### Sample size calculation

A sample size calculation was conducted to estimate the number of participants (nurses) required. Assuming a desired trial completion rate of 80% (*p* = 0.80) (see Table 3) and a maximum likely error of 10% with a 95% confidence level (*α* = 0.05), a required sample of 62 nurses was determined. This calculation was performed using R software. Our target sample size of 96 nurses accommodates the possibility of variations in cluster size, resulting from the availability of nursing staff at different sites.

### Recruitment

The study team will promote the study through flyers, word of mouth, and advertising in newsletters, webpages, and on various social media platforms via existing networks. Sites may contact the research team if they are interested in participating. The research team will also send invitation emails to residential aged care providers. Information sessions will be provided to potential participating sites which will be conducted either on site or online. If a residential aged care provider expresses interest in participating as a site, they will first be required to complete a demographic survey to determine their eligibility and site characteristics prior to randomisation. We acknowledge that this recruitment approach may result in a sample biased towards providers who are motivated to improve catheter care practices and are willing to allow researchers to monitor staff and organisational performance.

### Randomisation

An independent statistician at a separate institution will perform block randomisation, stratified according to three site sizes (small, medium, and large). Blocks will be randomly sized either two or four. The randomisation sequence will be generated in R. The National Ageing Research Institute (NARI) team will remain blinded to the allocation sequence and provide the statistician with numerical site study IDs and corresponding size values. The NARI research team will inform each aged care provider whether they have been allocated to the intervention group or control group following randomisation. As this study concerns the implementation of training resources vs usual care, it is not possible to blind the entire research team or sites to their group allocation. Only the Project Manager and three Research Assistants will have access to information about all sites’ group allocation. The rest of the research team, including the Chief Investigator and the statistician, will remain blinded to the identities of sites for the duration of the trial and subsequent statistical analysis of trial data.

### Intervention

Participants (managers/senior leaders, nurses, and PCWs) at intervention sites will access all elements of the *Bundle*. The *Bundle* will be implemented in two consecutive phases, (1) education phase and (2) application to practice phase.

### Phase 1: education

Phase 1 is designed to take account of managers’/senior leaders’, nurses’ and PCWs’ different roles and responsibilities. Education comprises an online catheter care course for nurses, an in-person catheterisation skills workshop for nurses delivered by CHA, an online catheter care course for PCWs, and a catheter care toolkit for managers and senior leaders.

#### The online catheter care course for nurses

The online catheter care course for nurses is self-paced and takes 5–8 h to complete. It comprises 5 modules addressing the following competencies: (1) emotionally supporting a person living with an IDC, (2) applying clinical decision making prior to urinary catheterisation, (3) understanding and applying principles of insertion and/or removal of a urethral or changing a suprapubic catheter, (4) knowing and implementing evidence-based nursing interventions to promote physical wellbeing, (5) minimising and troubleshooting common issues related to urinary catheterisation. The learning material was validated by advanced practice nurses.

#### The catheterisation skills workshop for nurses

The catheterisation skills workshop has been designed for nurses by CHA and will be delivered by facilitators who are advanced practice nurses who have advanced clinical skills or knowledge in continence or urology nursing. The workshop includes practice of indwelling catheterisation insertion on both male and female manikins as well as changing a suprapubic catheter. Workshop facilitators use a comprehensive checklist to assess learners and provide feedback on areas for development to each learner, based on their performance. The workshops will be conducted either on-site or at a nearby alternative venue if required. The number of nurses able to attend each workshop is limited to four due to the intensity of the program.

#### The online catheter care course for PCWs

Participating PCWs at the intervention sites will complete their own online course that consists of three learning modules that have been designed to take account of their roles and responsibilities in residential aged care homes. The learning material was validated by PCWs. We estimate it will take up to 1 h to complete each module. The content is interactive and includes a combination of text, short videos, quizzes, and scenario-based reflective exercises.

#### The catheter care toolkit for managers and senior leaders

The toolkit is a knowledge translation resource that is designed to support residential aged care managers and senior leaders to coordinate, supervise, and manage catheter care in the workplace. It includes the following resources to assist them to implement, evaluate and sustain the intervention:

- Information about the clinical risks associated with IDCs
- The components of the *Bundle*
- The knowledge and skills nurses and PCWs require to deliver person-centred catheter care
- A Catheter Care Audit Tool to assist self-assessment of their organisational capacity to deliver person-centred urinary catheter care.

Given the lack of evidence and guidance about IDC care in the residential aged care setting, we anticipate site management will require the information to influence the quality of catheter care.

### Phase 2: application to practice

Phase 2 runs for 3 months after participating staff from each site have completed the course and workshop. Three advanced practice nurses with expertise in catheter care will provide coaching support over 3 months. The level and frequency of support will be planned with each participant and will be influenced by their role, responsibilities, scope of practice, how many residents with IDCs they are caring for and their overall baseline experience managing IDCs. Coaching will be available on an individual basis and offered online and by phone, depending on the nurses’ and managers’/senior leaders’ individual preferences. The frequency will also be flexible and responsive to their preference.

### Comparator

Sites assigned to the control arm will continue with usual care during the trial period. After data collection is complete at their site, they will be given access to all the resources of the *Bundle* except the Catheterisation Skills Workshops for nurses about catheter insertion. If they wish to complete the workshop after the research has been concluded, they will be referred to CHA to discuss their individual needs.

### Adherence

Adherence to the intervention will be monitored by (i) online learning management system’s reporting and analytics features; (ii) workshop attendance records; and (iii) completion of data collection at each time point. Intervention fidelity will be assessed through qualitative semi-structured interviews with participants, workshop facilitators, and experts delivering the online/phone support.

### Outcome measures

#### Primary outcome

The primary outcome is the feasibility of implementing the intervention (*IDC-IMPROVE Catheter Care Bundle*) into Australian residential aged care homes. The following feasibility outcomes will be assessed as a composite primary outcome: (i) the acceptability of the *Bundle*, (ii) the fidelity of the implementation, (iii) the compatibility of the *Bundle* with standard aged care home IDC care.

Measures related to the feasibility and acceptability of both the trial and the *Bundle* are outlined in Table 1. Data collection timepoints are provided in Table 2. The feasibility of conducting a definitive cRCT is assessed against a priori progression criteria outlined in Table 3. If thresholds are not met, other qualitative data may be used to inform modifications of the *Bundle* and the trial design.

**Table 1:** Description of instruments, timepoints, and target groups for data collection

Timepoint	Intervention	Control	Instrument	Description
Site and participant demographic information				
t_0_	X	X	Aged care home demographic survey	Basic information on size, ownership and staff numbers
t_0_	X	X	Nurse/PCW Demographic Survey	Basic information on qualifications, years in nursing, years in aged care etc.
Clinical data collection tools				
t_0_, t_1_, t_2_	X	X	IDC-IMPROVE Nurses’ Catheter Care Knowledge and Confidence Scale (KCS-Nurse)	The KCS-Nurse is a newly developed scale that contains 51 items to measure nurses’ self-reported knowledge and confidence to deliver person-centred IDC care
t_0_, t_1_, t_2_	X	X	IDC-IMPROVE PCW Catheter Care Knowledge and Confidence Scale (KCS-PCW)	A newly developed scale that contains 34 items to measure PCWs’ self-reported knowledge and confidence to deliver person-centred IDC care
t_1_	X	X	Nurse Professional Competence (NPC) Scale Short Form	The NCP Scale-SF is a reliable and valid tool with 35-items that measures nurses’ self-reported competence in 6 key domains. Will be completed by nurses via survey. Findings to be correlated with the KSC-Nurse to establish the convergent validity of the KCS-Nurse
t_0_, t_1_	X	X	IDC-IMPROVE Clinical Data Extraction Form	Clinical information about age range, gender, IDC type, reason for IDC, duration of IDC, number of IDC changes, disciplinary background of staff who perform the change, number and type of IDC-related complications, and number of admissions to hospital and emergency departments for IDC-related complications for residents with an IDC for the prior 3 months, to be completed by managers/senior leaders or RNs
Feasibility and sustainability data collection tools				
t_0_, t_2_	X	X	IDC-IMPROVE Catheter Care Audit Tool	A pre and post intervention audit of each aged care home, IDC-related organisational policies and practices, to be completed by managers/senior leaders and administered with the aged care home demographic survey
t_0_, t_1_, t_2_	X		Participant Completion and Withdrawal Record	Number of nurses and PCWs who enrol, complete or withdraw from the education or application to practice phase
t_1_	X		Online Catheter Care Course for Nurses Feedback Survey	Rating scale to capture nurses’ satisfaction with the content and questions on their experience of the course
t_1_	X		Catheterisation Skills Workshop Feedback Survey	An online survey nurses will be asked to complete at the end of the catheterisation skills workshop
t_2_	X		Semi-structured qualitative interviews	Interviews will be conducted with nurses, PCWs and managers exploring participants’ perspectives and experiences with the *Bundle*, barriers and enablers to implementing the trial, contextual factors affecting IDC care and implementation of the *Bundle*

**Table 2:**
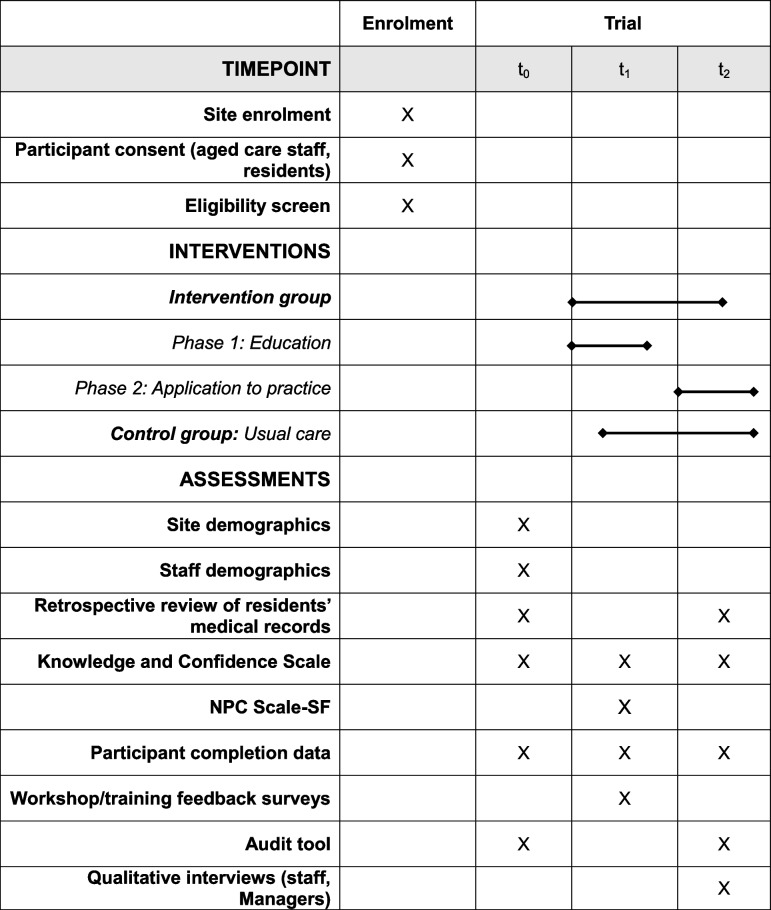
Timeline of study procedures, including enrolment, interventions, and assessment points

**Table 3:** Criteria, data collection methods, and thresholds for progression to a definitive trial

Criteria	Data collection	Threshold
(ii) Acceptability of the bundle	• Feedback surveys • Audit tool • Qualitative interviews	• 75% + participants “satisfied” or “very satisfied” • Thematic analysis of qualitative interviews • Descriptive analysis of audit tool
(iii) Fidelity of implementation	• Participant completion and withdrawal record • KCS-Nurse • KCS-PCW • NPC-Scale-SF • Clinical data extraction form	• Site retention: 20 sites complete all trial phases • Participant retention: ≥ 80% of enrolled participants complete all study follow-ups (t_0_, t_1_, t_2_) • Fidelity to data collection: 75% of all *expected* data points collected across KCS-Nurse, KCS-PCW, NPC-Scale-SF, and Clinical Data Extraction Forms from sites and participants who complete all trial phases • Internal Consistency of KCS-Nurse: Achieves acceptable internal consistency
(iv) Compatibility of bundle with standard practice	• Qualitative interviews • Audit tool	• Thematic analysis of qualitative interviews • Descriptive analysis of audit tool

Prior to site randomisation, information will be collected from organisations on their size, ownership and staffing, and from individual nurses and PCWs in terms of their qualifications, years in nursing and in working in residential aged care. The research team will provide sites with extraction forms to obtain key information from the medical records of residents with IDCs who consent to participate in the study. Research staff will be trained in obtaining informed consent and in the use of the extraction forms prior to inviting residents to participate. We anticipate some participating nurses may complete the online course but will be unable to complete a workshop due to workshop capacity limitations. These nurses will be flagged, and their outcome data will not be combined with workshop participants’ data.

#### IDC-IMPROVE Nurses’ Catheter Care Knowledge and Confidence Scale (KCS-Nurse)

All participating nurses will complete the KCS-Nurse scale at t_0_, t_1_, and t_2_. This scale was developed for this study to assess aged care nurses’ knowledge of, and confidence related to urinary catheters and catheter care. It comprises two sections: confidence and knowledge. The confidence section is theoretically informed by five key competency domains: (1) ability to psychologically support a person living with a long-term urinary catheter; (2) ability to apply clinical decision-making prior to urinary catheterisation; (3) understanding and application of principles for inserting and/or removing a urethral or suprapubic catheter; (4) knowledge and application of evidence-based nursing interventions to promote physical well-being; and (5) ability to minimize and troubleshoot common issues related to long-term urinary catheterisation. This section includes 21 statements regarding nursing actions specific to catheter care and uses a 5-point Likert scale (ranging from 0 for “no confidence” to 5 for “high confidence”) to capture responses; the highest possible score for these 21 statements is 105. The knowledge section includes 30 items with a dichotomous measurement scale, where a correct answer scores 1 and an incorrect or uncertain answer scores 0, resulting in a highest possible score of 30. A survey of 11 experts conducted by the research team has already demonstrated strong content validity (S-CVI/Ave = 0.97). Internal validity, construct validity, and convergent validity of the scale will be estimated in this study.

#### IDC-IMPROVE Personal Care Worker Catheter Care Knowledge and Confidence Scale (KCS-PCW)

All participating PCWs will complete the KCS-PCW scale at t_0_, t_1_, and t_2_. The scale was developed for this study to assess PCWs’ confidence and knowledge regarding urinary catheters and catheter care. The confidence section includes 14 statements encompassing four domains: (1) ability to emotionally support a person living with a urinary catheter; (2) understanding of the basic structure and function of the lower urinary tract; (3) understanding of the purpose, main types, and reasons why a person may need a urinary catheter; and (4) knowledge of the role of the personal care worker caring for a person living in a residential aged care home who has a urinary catheter. This section uses a 5-point Likert scale to capture responses, ranging from zero (no confidence) to five (high confidence), with the highest possible score for the 14 statements being 70. The knowledge section consists of 20 items with a dichotomous measurement scale where a correct answer scores 1 and an incorrect answer scores 0, resulting in a highest possible score of 20.

### Secondary outcomes

The secondary outcomes include data likely to be used in a larger definitive trial to test the effectiveness of the intervention. The compatibility of the *Bundle* with current residential aged care catheter care practices, procedures and policies will be assessed through a pre- and post-intervention audit of each residential aged care home IDC-related organisational policies and practices, as well as post-intervention semi-structured interviews with managers/senior leaders about their organisational IDC policies and practices.

Pilot data on the preliminary effects of the *Bundle* on rates of IDC-related complications will be assessed through a pre- and post-retrospective review of medical records of residents with an IDC. Records will be reviewed for incidences of CAUTI (symptomatic and asymptomatic), catheter blockages, catheter-related trauma, leakage/bypassing, and hospitalisations related to catheter complications. Pilot data on the effects of the *Bundle* on nurses’ knowledge and confidence providing IDC care will be assessed through the *Nurses’ Catheter Care Knowledge and Confidence Scale* (KCS-Nurse), a study-specific survey of nurses’ knowledge and confidence.

### Data analysis

Feasibility outcomes will be analyzed using quantitative and qualitative approaches. The R software program will be used for all quantitative data manipulation and analysis. Descriptive analysis will be conducted for all demographic and scaled outcomes, reporting at baseline (t_0_), post-intervention (t_1_), and follow-up (t_2_) timepoints. The KCS-Nurse scores will be assessed for normality using descriptive statistics and histograms. All subscale scores will be analysed using the median with the 25th and 75th percentiles (IQR), and normally distributed scores will be additionally analysed with means and 95% confidence intervals (CI). Discrete and categorical data will be analysed using frequencies with relative frequencies. Descriptive analyses will be assessed according to progression criteria and reported against the prescribed thresholds in Table 3.

Survey feedback will be summarised. Qualitative interview data will be recorded, transcribed verbatim, checked for accuracy, and analysed using thematic analysis [21]. Two members of the research team will analyse the data independently and conduct a thematic analysis. Qualitative findings will be used alongside the analysis of progression criteria and feedback from surveys to conduct a process evaluation. This will explore how the intervention was experienced by participants and any changes that they think would improve the trial and intervention and their implementation. A “Framework” analytical approach [22] informed by the 14-domain version of the Theoretical Domains Framework [23] and based on the research objectives will be used to describe the feasibility of the *Bundle*.

The internal consistency, construct validity and convergent validity of the KSC-Nurse will be analysed. Internal consistency will be estimated using Cronbach’s alpha. Construct validity will be estimated using confirmatory factor analysis (CFA), based on the 5 key competency domains of the scale. The model fit will be assessed using the Comparative Fit Index (CFI). Convergent validity will be estimated by calculating the Pearson correlation coefficient between the KSC-Nurse and the Nurse Professional Competence Scale-Short Form (NPC Scale-SF) [24], accounting for clustering. The intra-cluster coefficient (ICC) will be calculated based on KSC-Nurse scores to explore clustering effects and inform implementation of the instrument in other studies.

### Oversight and monitoring

A Project Steering Committee has been established, consisting of all investigators and partners. They meet regularly to review and monitor the progress of the Project to ensure milestones are met, and to identify potential risks and unanticipated events. The Project Manager (employed by NARI) will coordinate the day-to-day administrative aspects of the study and the implementation of the protocol under the direction of the Project Steering Committee.

### Data management

Study data will be stored on the NARI internal file server in a password protected folder, except for the randomisation sequence, which will be securely stored by the statistician on the University of Melbourne networked storage space. Sites will record data into REDCap, hosted on the NARI server. Data will be extracted into Excel spreadsheets for checking and cleaning. Anonymised data will be securely transferred via MS OneDrive to the independent statistician for analysis using R. Upon completion of the study, all documentation and data will be archived for 5 years on the NARI server, then securely destroyed.

## Discussion

Up to 8% of people admitted to residential aged care have catheters, yet there are inconsistent approaches to IDC care, with many residential aged care nurses feeling unprepared and disempowered to provide routine catheter care and to prevent or manage complications [24, 25]. The IDC-IMPROVE Project aims to support the residential aged care workforce through training to identify, prevent and manage IDC-related problems and improve IDC care in residential aged care homes. For nurses, this includes how to perform an uncomplicated routine catheter change onsite, and for PCWs, to know the fundamentals of caring for someone with an IDC and escalating any signs and symptoms of complications to nurses or nurse leaders. Most IDC-related research and training to date, including the implementation of catheter care bundles, have been developed for hospital contexts [26, 27]. These have a predominantly acute care focus and typically assume IDCs are only short term. Managing long-term catheterisation in residential aged care homes should prioritise a person-centred approach that upholds the dignity and privacy of residents. If successful, the IDC-IMPROVE Project has the potential to create new knowledge and provide one of the first evidence-based interventions to improve IDC-care in residential aged care contexts in Australia.

This study will provide new knowledge of the feasibility of conducting a cRCT to improve catheter care in a residential aged care setting, including acceptability of the intervention itself and fidelity to trial procedures. The process evaluation will generate new knowledge about current practices related to catheter care in residential aged care homes in Australia, as well as barriers and enablers to implementing a trial designed to facilitate practice change.

## Supplementary Information

Supplementary Material 1

## Data Availability

The datasets obtained and/or analysed during the current study are available from the corresponding author on reasonable request.

## Abbreviations

IDC: Indwelling urinary catheter
CAUTI: Catheter-associated urinary tract infection
CHA: Continence Health Australia
CI: Confidence interval
cRCT: Cluster randomised control trial
EAUN: European Association of Urological Nurses
EN: Enrolled nurse
ICC: Intra-cluster correlation coefficient
IQR: Inter-quartile range
KCS-Nurse: Nurses’ Catheter Care Knowledge and Confidence Scale
MRFF: Medical Research Future Fund
NARI: National Ageing Research Institute
NHMRC: National Health and Medical Research Council
NPC Scale SF: Nurse Professional Competence Scale Short Form
PCW: Personal care worker
RN: Registered nurse

## Footnotes

1 The terms “residential aged care” and “aged care home” reflect usage in Australian legislation (Aged Care Act 1997), which is defined as personal care or nursing care specifically for older people, delivered in a residential facility.
